# Low-intensity pulsed ultrasound therapy promotes recovery from stroke by enhancing angio-neurogenesis in mice in vivo

**DOI:** 10.1038/s41598-021-84473-6

**Published:** 2021-03-02

**Authors:** Sadamitsu Ichijo, Tomohiko Shindo, Kumiko Eguchi, Yuto Monma, Takashi Nakata, Yoshihiko Morisue, Hiroshi Kanai, Noriko Osumi, Satoshi Yasuda, Hiroaki Shimokawa

**Affiliations:** 1 Department of Cardiovascular Medicine, Tohoku University Graduate School of Medicine, 1-1 Seiryo-machi, Aoba-ku, Sendai, 980-8574 Japan; 2 Department of Developmental Neuroscience, Tohoku University, Sendai, Japan; 3 Biomedical Engineering for Health and Welfare, Graduate School of Biomedical Engineering, Tohoku University, Sendai, Japan; 4 Department of Electronic Engineering, Tohoku University Graduate School of Engineering, Sendai, Japan; 5 International University of Health and Welfare, Narita, Japan

**Keywords:** Neuro-vascular interactions, Neurogenesis, Stroke, Angiogenesis

## Abstract

Since the treatment window of thrombolytic therapy for stroke is limited, new therapy remains to be developed. We have recently developed low-intensity pulsed ultrasound (LIPUS) therapy to improve cognitive dysfunction in mouse models of vascular dementia and Alzheimer’s disease. Here, we further aimed to examine whether our LIPUS therapy improves neurological recovery from ischemic stroke, and if so, to elucidate the mechanisms involved. In a mouse model of middle cerebral artery occlusion (MCAO), we applied LIPUS (32 cycles, 193 mW/cm^2^) to the whole brain 3 times in the first week (days 1, 3, and 5) after MCAO. We evaluated neurological functions using behavioral tests and performed histological analyses. Furthermore, to elucidate how LIPUS works within the injured brain, we also tested the effects of LIPUS in endothelial nitric oxide synthase (eNOS)-deficient (eNOS^−/−^) mice. In wild-type mice, the LIPUS therapy markedly improved neurological functions in the tightrope and rotarod tests at 28 days after MCAO. Histological analyses showed that the LIPUS therapy significantly increased the numbers of CD31-positive blood vessels in the perifocal lesion and doublecortin (DCX)-positive neurons in the ischemic striatum, indicating the angio-neurogenesis effects of the therapy. Importantly, these beneficial effects of the LIPUS therapy were totally absent in eNOS^−/−^ mice. No adverse effects of the LIPUS therapy were noted. These results indicate that the LIPUS therapy improves neurological functions after stroke through enhanced neuro-angiogenesis in mice in vivo in an eNOS-dependent manner, suggesting that it could a novel and non-invasive therapeutic option for stroke.

## Introduction

Stroke is the leading cause of death and disability associated with a limited degree of functional recovery^1^. With the progress of medical technology in the past decades, increasing number of stroke patients survived from the initial injury. Consequently, 60% of survivors have disabilities in the arm or leg, and up to one third of them need to stay in a nursing home or to use assistant devices for independent living^2,3^. Thus, it is crucial to develop effective treatment for stroke patients. Brain neuro-restoration leads to considerable post-stroke functional recovery. The brain attempts to repair itself after ischemic stroke by neurogenesis and angiogenesis^4^. Neural stem/progenitor cells (NPCs) and endothelial progenitor cells (EPCs) play important roles in neurogenesis and angiogenesis, respectively^5^. After stroke, NPCs migrate to the ischemic boundary where angiogenesis takes place, which is closely associated with cerebral vessels in neuro-vascular unit. Indeed, suppression of angiogenesis substantially reduces migration of newly formed NPCs to the ischemic region^5^, and activated endothelial cells (ECs) secrete vascular endothelial growth factor (VEGF) to increase neurogenesis^5^.

Among several ultrasound technologies, low-intensity pulsed ultrasound (LIPUS) has emerged as a non-invasive therapy for several diseases. We have previously demonstrated that the LIPUS therapy with specific pulsed conditions has therapeutic effects for ischemic and pressure-overloaded heart disease in animal models^6–8^. We found that the LIPUS therapy upregulated neurotrophins, including VEGF and endothelial nitric oxide synthase (eNOS)^6–8^. Since endothelium-derived NO exerts a variety of beneficial cardiovascular effects, activation of eNOS could be an effective therapeutic strategy for cardiovascular disease^9,10^. Furthermore, we also have recently demonstrated that the LIPUS therapy improves cognitive dysfunction in mouse models of vascular dementia and Alzheimer’s disease through enhancement of eNOS upregulation^11^. However, the effects of the LIPUS therapy on stroke remain to be examined.

In the present study, we thus examined whether the LIPUS therapy can promote post-stroke functional recovery through angiogenesis and neurogenesis, and if so, to elucidate the mechanisms involved.

## Results

### LIPUS therapy improves functional recovery following MCAO

**Figure 1:**
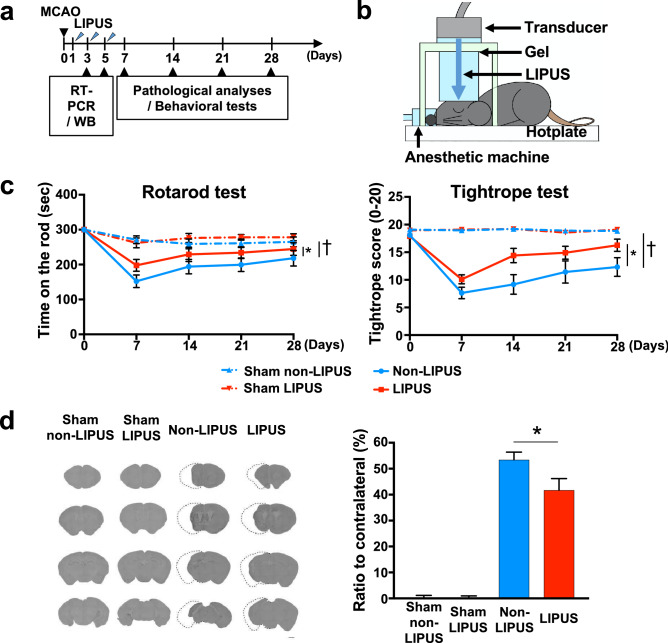
Whole-brain LIPUS therapy improves functional recovery after MCAO. (**a**) The timeline of the experiments. (**b**) Schematic drawing showing the application of whole-brain LIPUS in mice. (**c**) Tightrope and rotarod tests were performed on days 7, 14, 21, and 28 following MCAO or sham surgery; n = 10. *P < 0.05 (C; two-way ANOVA, D; Student’s *t*-test), ^†^*P* < 0.05 (two-way ANOVA). (**d**) Cresyl violet staining was performed on day 28 following MCAO or sham surgery; n = 3 (sham), n = 8 (MCAO). The scale bar represents 100 μm. *P < 0.05 (Student’s *t*-test).

In the middle cerebral artery occlusion (MCAO) model in mice, the LIPUS therapy (Fig. 1a,b) had no effects on body weight or systolic blood pressure (*P* = 0.454, *P* = 0.971, Fig. S1). The LIPUS group showed no increased events of cramps or cerebral hemorrhage compared with the Non-LIPUS group, which underwent the same procedure but without the LIPUS therapy (data not shown). The LIPUS therapy markedly improved neurological functions compared with the Non-LIPUS group in the rotarod and in the tightrope tests (rotarod test, *P* = 0.012; tightrope test, *P* < 0.001, Fig. 1c, Movie S1, Movie S2). There were no significant differences in the corner or the Barnes circular maze test between the 2 groups (Fig. S3). Moreover, the LIPUS therapy significantly reduced the infarct size at day 28 compared with the Non-LIPUS group (*P* = 0.045, Fig. 1d).

### LIPUS therapy promotes angiogenesis around ischemic lesion

**Figure 2:**
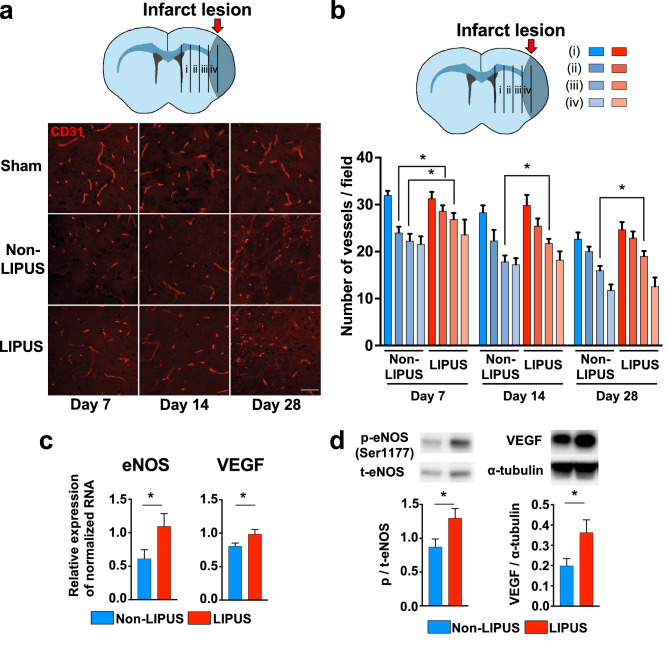
The LIPUS therapy promotes angiogenesis and upregulates angiogenesis- related molecules. (**a**) Schematic drawing showing the ischemic hemisphere section and 4 regions related with the lesion at (i) 40–250 μm, (ii) 250–500 μm, (iii) 500–750 μm, and (iv) 750–1000 μm from the SVZ. Representative images of ischemic hemisphere sections at 500–750 μm from the SVZ immunostained for CD31 (endothelial cell marker) on days 7, 14, and 28. The scale bar represents 50 μm. (**b**) Schematic drawing showing 4 lesions of interest at different distances from the SVZ. The numbers of CD31-positive vessels in the indicated lesions at days 7, 14, and 28 (n = 8 per group). (**c**) RT-qPCR analysis of eNOS and VEGF at day 3 (n = 14 per group). (**d**) Representative Western blot and quantification of phosphorylated eNOS at Ser1177 and VEGF at day 5 (n = 10 per group). *P < 0.05 (Student’s *t*-test). Uncropped blots are shown in Fig. S10.

Next, to examine whether the LIPUS therapy increases vascular density in the MCAO model, we examined CD31-positive vessel density in the injured striatum (Fig. 2a). In the normal striatum areas, including the subventricular zone (SVZ), there was no difference in CD31-positive vessels between the 2 groups. However, in the ischemic lesion, the number of CD31-positive vessels, especially at 250 ~ 750 μm from the lateral ventricle, was significantly increased in the LIPUS group compared with the non-LIPUS group at day 7 (250–500 μm, *P* = 0.019; 500–750 μm, *P* = 0.039) (Fig. 2b). Moreover, the number of CD31/Ki67 double positive cells at 500–750 μm from the lateral ventricle at day 7 was significantly increased in the LIPUS group compared with the Non-LIPUS group (*P* = 0.029, Fig. S4c), suggesting that the LIPUS therapy induced angiogenesis in the MCAO model. This angiogenic effect of the LIPUS therapy was noted until day 28 (Fig. 2b).

### LIPUS therapy upregulates angiogenic factors

In order to elucidate the molecular mechanisms of the beneficial effects of the LIPUS therapy on angiogenesis, we examined the expressions of mRNA and proteins that are related to angiogenesis. mRNA expression levels of eNOS, VEGF, and fibroblast growth factor (FGF) were significantly higher in the LIPUS group compared with the Non-LIPUS group (eNOS, *P* = 0.047; VEGF, *P* = 0.041; FGF, *P* = 0.021, Fig. 2c, Fig. S5a). The protein levels of eNOS phosphorylated at Ser1177, VEGF, and extracellular signal-regulated kinase (ERK) phosphorylated at Thr202/Tyr204 were higher in the LIPUS group compared with the Non-LIPUS group (eNOS, *P* = 0.031; VEGF, *P* = 0.034; ERK, *P* = 0.030, Fig. 2d, Fig. S5b). In contrast, mRNA expression level of angiopoietin-1 was comparable between the 2 groups (Fig. S5a).

### LIPUS therapy promotes neurogenesis

**Figure 3:**
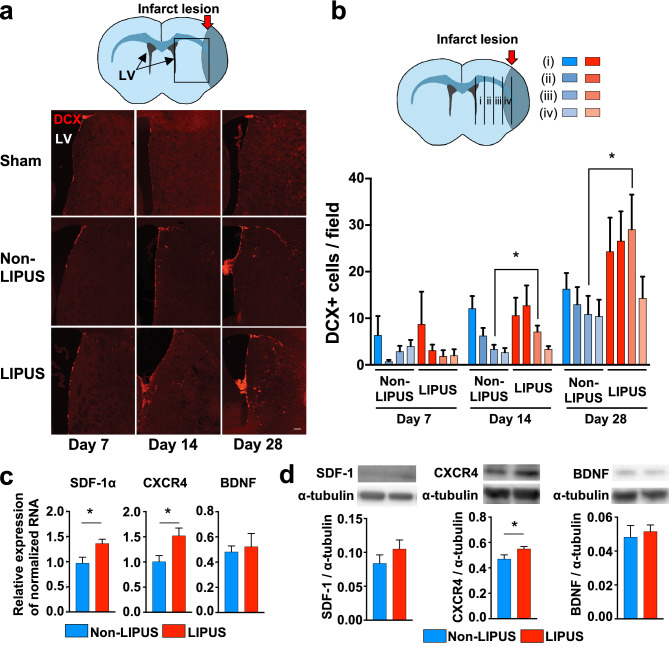
The LIPUS therapy promotes neurogenesis and upregulates the molecules related to neuronal migration. (**a**) Schematic drawing showing the coronal section of the mouse brain and lesion areas of interest. Representative images of ischemic hemisphere sections immunostained for DCX on days 7, 14, and 28. The scale bar represents 100 μm. (**b**) Schematic drawing showing the ischemic hemisphere section and 4 regions related with the lesion at (i) 40–250 μm, (ii) 250–500 μm, (iii) 500–750 μm, and (iv) 750–1000 μm from the SVZ. The numbers of DCX-positive cells in the indicated regions at day 7, 14, and 28 (n = 8 per group). (**c**) RT-qPCR analysis of SDF-1, CXCR4, and BDNF at day 3 (n = 14 per group). (**d**) Representative Western blot and quantification of SDF-1, CXCR4 and BDNF at day 5 (n = 10 per group). *P < 0.05 (Student’s *t*-test). Uncropped blots are shown in Fig. S10.

To examine whether the LIPUS therapy promotes neurogenesis after MCAO, we examined the number of cells expressing the immature neuronal marker, doublecortin (DCX), in the striatum. Although there was no significant difference in DCX-positive cells at day 7, more DCX-positive cells were noted in the ischemic striatum of the LIPUS-treated group compared with the Non-LIPUS group at days 14 and 28 (500–750 μm at day 14, *P* = 0.033; 500–750 μm at day 28, *P* = 0.042, Fig. 3a,b).

### LIPUS therapy increases protein levels of factors related to neuronal migration

In order to elucidate the molecular mechanisms of the beneficial effects of the LIPUS therapy on neurogenesis, we then examined mRNA and protein expressions of neurogenesis-related factors. We found that mRNA expression levels of stromal cell-derived factor (SDF)-1α and C-X-C motif chemokine receptor 4 (CXCR4) were significantly higher in the LIPUS group compared with the Non-LIPUS group (SDF-1α, *P* = 0.012; CXCR4, *P* = 0.013; Fig. 3c), which also was the case for protein level of CXCR4 (*P* = 0.044, Fig. 3d). In contrast, mRNA and protein levels of brain-derived neurotrophic factor (BDNF) were comparable between the Non-LIPUS and LIPUS groups (Fig. 3c,d). The protein levels of SDF-1 and phosphorylated Akt at Ser 473 were also unchanged between the two groups (Fig. 3d, Fig. S5b).

To examine cellular source of SDF1 and CXCR4, we performed double immunofluorescence staining for both CD31-positive and DCX-positive cells. SDF-1 and CXCR4 were expressed in CD31-positive cells at day 7 (Fig. S6a,b). In contrast, few DCX-positive cells expressed SDF-1 at day 7 (Fig. S7a), and CXCR4 was expressed only in a few DCX-positive cells (Fig. S7b). These results suggest that CD31-positive cells contributed to the production of SDF-1 and CXCR4 and migration of DCX positive cells.

### Essential role of eNOS in the beneficial effects of the LIPUS therapy

**Figure 4:**
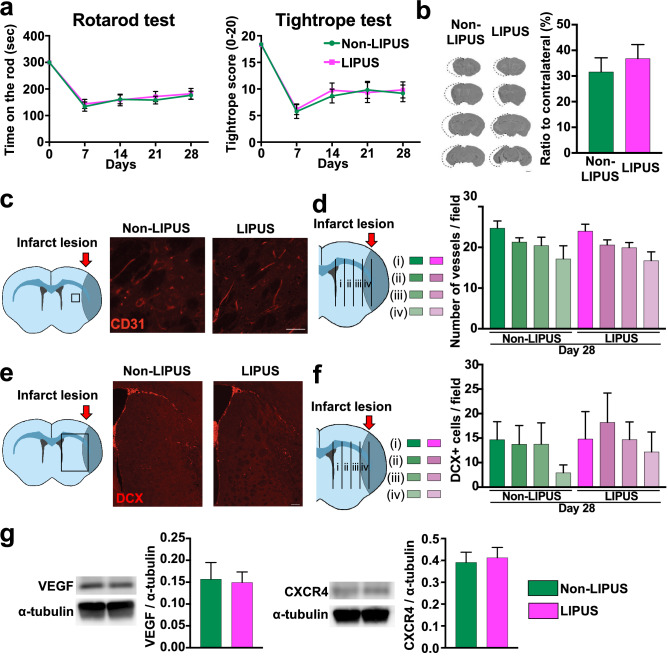
Crucial role of eNOS for the beneficial effects of LIPUS in the MCAO model. (**a**) Behavioral tests were performed on days 7, 14, 21, and 28 in the Non-LIPUS and LIPUS-treated eNOS^−/−^ mice subjected to MCAO (n = 6 per group). (**b**) Cresyl violet staining was performed on day 28 following MCAO (n = 6 per group). (**c**) Representative images in the peri-infarct lesion immunostained for CD31 on day 28. The scale bar represents 50 μm. (**d**) The number of CD31-positive vessels at (i) 40–250 μm, (ii) 250–500 μm, (iii) 500–750 μm, and (iv) 750–1000 μm from the SVZ on day 28 (n = 6 per group). (**e**) Representative images of the ischemic striatum immunostained for DCX on day 28. The scale bar represents 100 μm. (**f**) The numbers of DCX-positive cells at (i) 40–250 μm, (ii) 250–500 μm, (iii) 500–750 μm, and (iv) 750–1000 μm from the SVZ at day 28 (n = 6 per group). (**g**) Western blot analysis of VEGF and CXCR4 on day 5 (n = 6 per group). Uncropped blots are shown in Fig. S10.

Our previous study showed that up-regulation of eNOS is crucial for the angiogenic effects of the LIPUS therapy. Indeed, in the present study, the protein level of phosphorylated eNOS was significantly higher in the LIPUS group compared with the Non-LIPUS group. To evaluate the role of eNOS in the LIPUS-induced effects, we next examined the influence of eNOS ablation in the MCAO model using eNOS-deficient (eNOS^−/−^) mice^12^. Importantly, in eNOS^−/−^ mice, the LIPUS therapy had no effect on MCAO-induced behavior outcome, infarct size, CD31-positive vessel density, or the number of DCX-positive neuroblasts (Fig. 4a–f). Furthermore, the expression levels of neurotrophins related to angiogenesis (VEGF) and neurogenesis (CXCR4 and ERK) after MCAO were not increased in LIPUS-treated eNOS^−/−^ mice (Fig. 4g, Fig. S8). Thus, eNOS is crucial for the beneficial effects of the LIPUS therapy in the MCAO model.

### LIPUS therapy does not improve functional recovery following MCAO when used in the chronic phase

Finally, we assessed whether the LIPUS therapy improves neurological functions after MCAO when used in the chronic phase. In this experiment, the LIPUS therapy was started 28 days after MCAO (Fig. S9a). We performed behavioral tests at days 28, 56, 84 and 112. However, there were no significant differences between the LIPUS and the Non-LIPUS groups in the rotarod, tightrope, or corner test (Fig. S9b). This may be because maturation of blood vessels induced by angiogenic stimuli is completed within 28 days in mice^13^, and even if the angiogenesis is stimulated thereafter, the tissues are unable to respond.

## Discussion

Cerebral infarction is a disease with poor prognosis that significantly impairs activities of daily living (ADL) due to paralysis of the extremities of the patients. We have demonstrated that the LIPUS exerts angiogenic and anti-inflammatory effects and have developed the LIPUS therapy for the treatment of ischemic heart disease first^6,7^ followed by that for dementia^11^. Thus, in the present study, we aimed to examine whether the whole-brain LIPUS therapy is also effective for the treatment of ischemic stroke in mice in vivo, and if so, to elucidate the mechanisms involved.

### LIPUS promotes angiogenesis and subsequent neurogenesis

**Figure 5:**
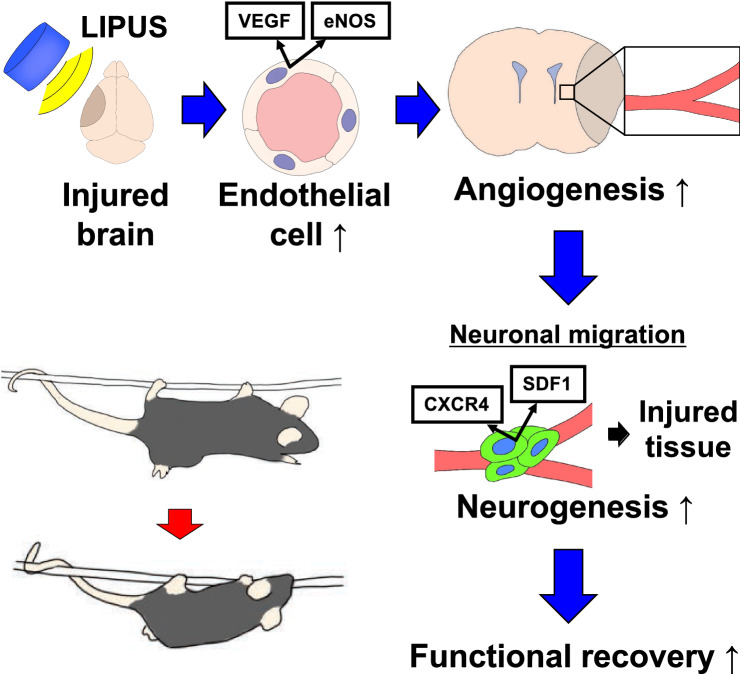
Schematic diagram showing the proposed mechanisms underlying the beneficial effects of whole-brain LIPUS in the MCAO model in mice. The present study demonstrates that the LIPUS therapy promotes functional recovery in MCAO mouse model, and that the beneficial effects of the LIPUS therapy depend on endothelial NO synthase (eNOS). Both angiogenesis and neurogenesis were promoted by the LIPUS therapy sequentially in this order, indicating that VEGF and eNOS-induced angiogenesis subsequently upregulates neurotrophins, including SDF-1, and CXCR4, and these molecules mediate neuronal proliferation and migration. Additionally, increased vessel density supports neuronal migration structurally. Finally, neuronal functional recovery can be improved by differentiated neural stem cells.

We have previously reported that the LIPUS therapy mainly acts on vascular endothelial cells, inducing angiogenesis in ischemic tissue through upregulation of eNOS^6–8,11^. In the present study, we consistently observed increased vessel density in the peri-infarct lesion associated with upregulation of VEGF and eNOS in the LIPUS group as we observed in the mouse model of vascular dementia^11^. During the recovery process, the LIPUS therapy induced more CD31-positive blood vessels, followed by DCX-positive neurons. This implies that neovessels generated by angiogenic effects of LIPUS induced neurogenesis and neuronal migration to the infarct lesion. Furthermore, we also confirmed a significant increase in the expressions of SDF-1α and CXCR4, which are known as molecules that regulate cell proliferation and cell migration^14^. It has been reported that CXCR4 is expressed in NPCs and the actions of SDF-1/CXCR4 complex are crucial for NPCs migration to SDF-1-riched injury sites after cerebral ischemia^15–17^. Thus, it is highly possible that the LIPUS therapy can enhance proliferation of neural stem cells and support their migration by creating suitable vasculature as a scaffold (Fig. 5).

### First evidence of the LIPUS treatment for clinical medicine

A few studies have previously attempted to apply focused LIPUS for the treatment of stroke in rodent models^18–20^. Although it has been reported that focused LIPUS applied to the ischemic site of brain directly induces angiogenesis^19^, our whole brain irradiation may be advantageous as it stimulates not only the ischemic border zone but also the SVZ known as neurogenic niche. Additional advantage of our whole-brain LIPUS therapy over the focused LIPUS therapies is that it does not require an expensive MRI machine and is easy to implement in clinical practice. Actually, our whole-brain LIPUS using convenient equipment is already in the phase 2 of the dementia trial (UMIN000033071). Moreover, the focused LIPUS has been applied either before or immediately after the injury^18–20^, whereas our whole-brain LIPUS was applied 1, 3, and 5 days after the injury, which may be more suitable for future clinical application.

### Study limitations

Several limitations should be mentioned for the present study. First, we did not analyze the function of individual brain cells, such as astrocytes, microglia, and other types of cells, for neuronal recovery after stroke. For example, LIPUS stimulates astrocytes and upregulates their BDNF production^21^. Other types of cells (e.g. oligodendrocyte precursor cells) may also have important roles in the beneficial effects of the LIPUS therapy on post-stroke neuronal recovery. Second, to confirm that newborn neurons are incorporated into the surrounding tissue and work functionally, differentiation, axonal outgrowth, and synaptogenesis should be analyzed. Further studies are needed to elucidate the mechanism of the beneficial effects of the LIPUS therapy on post-stroke functional recovery.

## Conclusions

The LIPUS therapy could be a novel treatment with minimal invasiveness, which may potentially be applied to a huge number of patients with stroke in an aging society. In the current clinical medicine, treatment options for ischemic brain injury are not sufficient, and there is no treatment for promoting neurogenesis after stroke. Thus, our whole-brain LIPUS therapy could be a novel treatment option not only for ischemic stroke but also for other neurodegenerative diseases, such as Parkinson disease, amyotrophic lateral sclerosis, spinocerebellar degeneration in future.

## Methods

All procedures were performed in accordance with the protocols approved by the Institutional Committee for the Use and Care of Laboratory Animals of Tohoku University (2017Ikumikae-174, 2017Idou-266), and the Guide for the Care and Use of Laboratory Animals published by the US National Institutes of Health (NIH Publication No. 85-23, revised 1985). The experimental timeline is shown in Fig. 1a.

### Mice

Male C57BL/6 mice (10–12 weeks-old, weight 22 to 28 g) and eNOS-deficient (eNOS^−/−^) mice (10–12 weeks-old, on a C57BL/6 background; weight 22 to 28 g) were used. eNOS^−/−^ mice were originally provided by P. Huang (Harvard Medical School, Boston, MA)^12^ and were backcrossed to C57BL/6 mice more than 10 generations. All mice were housed in a temperature-controlled (20 ± 2 °C) and humidity-controlled (60%) room. The number of animals used in each experiment is shown in Table S1.

### Induction of transient focal cerebral ischemia

Cerebral ischemia was induced using the intraluminal middle cerebral artery occlusion (MCAO) model as described previously^22,23^. Mice were anesthetized with 1.1% isoflurane (Wako Pure Chemical Industries, Ltd., Osaka, Japan) and were placed on a heating pad to prevent hypothermia; body temperature was maintained at 36.5 to 37.5 °C. MCAO was induced using a commercially available suture (602312PK10Re, Doccol Corp., MA, USA), which was withdrawn after 45 min of ischemia to allow reperfusion of the middle cerebral artery. Regional cerebral blood flow was measured with a laser-Doppler flowmetry (Omegawave, Tokyo, Japan) to confirm the induction of ischemia and reperfusion (Fig. S2). Animals that did not show less than 30% reduction of regional cerebral blood flow from pre-ischemia baseline levels during MCAO were excluded from further experimentation. All animals were randomly assigned to each treatment groups.

### Whole-brain LIPUS therapy

The experimental timelines are shown in Fig. 1a. The LIPUS therapy was performed as previously described, with slight modifications^11^. The LIPUS group was subjected to the LIPUS therapy 3 times in the first week (1, 3, and 5 days after MCAO), while the Non-LIPUS group underwent the same procedures including anesthesia but without the LIPUS treatment (Fig. 1a). For the LIPUS therapy, we used a planer ultrasound probe (HONDA ELECTRONICS, Co., Aichi, Japan). The LIPUS therapy was performed using the following conditions; center frequency = 0.5 MHz, pulse repetition frequency = 780 Hz, number of cycles = 32 (64-μsec burst length), and spatial peak temporal average intensity = 193 mW/cm^2^ ^11^. While in the previous study, the center frequency was 1.875 MHz^11^, we used in the present study 0.5 MHz for the purpose of effectively penetration through the skull. Additionally, in order to apply the LIPUS therapy for humans in future clinical trials, we have changed the shape of the transducer from the sector-type to the convex-type, which has already been used in our clinical trial for patients with Alzheimer’s disease (UMIN000033071). As in the previous study^11^, we used whole brain irradiation, and we applied LIPUS three times in the same position. Whole-brain irradiation is used to cover a wider range of lesions than the focused-ultrasound. The mouse’s head was shaved, and the LIPUS was applied to the head via an agar phantom gel under inhalation anesthesia with 0.5–1.0% isoflurane (Fig. 1b). The treatment was performed three times a day with each duration of 20 min.

### Behavioral tests

Behavioral tests were conducted on days 7, 14, 21, 28 following MCAO or sham surgery (Fig. 1a). The tests were conducted by researchers who were blinded with respect to whether the mouse received the LIPUS therapy or not.

#### Rotarod test

The Rotarod test was performed with MK-630B (Muromachi Kikai, Co., Tokyo, Japan) as previously described^24^. The cylinder speed was slowly increased from 4 to 40 rpm. Maximum speed was achieved after 260 s, and maximum testing time was 300 s. Mice were put on the cylinder, and the time spent on the device was determined. Mice were trained for 5 days before MCAO. The mean duration on the device was recorded with 2 measurements.

#### Tightrope test

Animals were placed on a 60 cm-long tightrope grasping the string with their forepaws according to a modified protocol from Doeppner et al.^24^. All animals were tested twice per time point. Maximum test time was 60 s, and results were scored from 0 (minimum) to 20 (maximum) according to a validated score (Table S2), depending on the time that animals spent on the rope and whether or not they reached to the platform.

#### Corner test

The corner test was used to evaluate sensorimotor asymmetry, as described previously^25^. The mice were placed in a corner with an angle of 30°. To exit the corner, the mouse could turn to the left or right and this choice was recorded. Although healthy animals leave the corner without side preference, mice suffering from stroke preferentially turn to the left, non-impaired body side^25^. The test was repeated 10 times, and the percentage of left turnings was calculated. Only turns involving full rearing along either wall were included.

#### Barnes circular maze test

We performed the Barnes task on “dry land,” a gray acrylic platform (122 cm in diameter), with 16 equidistant holes around the perimeter (O’Hara & Co., Tokyo, Japan). An overhead camera assessed the performance of mice as previously reported^26^. In the 3-day training period, 3 trials were performed per day, with a maximum trial length of 300 s. Each trial began with the start box positioned at the center of the maze and a mouse placed inside it for 30 s; the mouse was then permitted to explore the maze freely. After reaching the target and before being returned to its home cage, the mouse was left in the escape box for 30 s. If the mouse did not enter the escape box within 300 s, the researcher gently picked it up and placed it inside the target box for 30 s before returning it to its home cage. We assessed the latency (time to reach the target box) and compared the results between sham animals and the MCAO groups with or without the LIPUS therapy.

### Histological analyses

To assess the acute and chronic phase effects of the LIPUS therapy in the MCAO model in mice, histological analyses were performed on days 7, 14, and 28 after the procedure (Fig. 1a). Under deep anesthesia with isoflurane, the mouse was perfused with 4% paraformaldehyde, the brain was removed, fixed in 4% paraformaldehyde for 48 h, cryoprotected in 30% sucrose, and 10-μm coronial sections were cut using a cryostat (LEICA, Wetzlar, Germany). Cresyl violet staining was performed to evaluate infarct area^27–29^. The tissue defect in each experimental group was defined as the lesion, and the area of this defect was calculated by subtracting the area of the ipsilateral hemisphere from the area of the contralateral hemisphere. Each area was estimated by the ImageJ software (ImageJ version 1.52, National Institutes of Health, MD, USA; available at: http://imagej.nih.gov/ij/). The lesion volume was calculated from the sum of lesion areas of the 4 serial slices, spaced 2-mm apart. The percentage of the lesion volume presented in this study was calculated by dividing lesion volume by the volume of the contralateral hemisphere.

For immunofluorostaining, the sections were blocked with PBS containing 5% goat serum (Invitrogen, CA, USA) and 0.3% Triton X-100, and then incubated with primary antibodies at 4 °C overnight. After washing with PBS, sections were incubated with secondary antibodies conjugated with Alexa Fluor 488 (1:500, Thermo-Fisher Scientific, Waltham, MA, USA), Alexa Fluor 564 (1:500, Thermo-Fisher Scientific, Waltham, MA, USA) or Cy3 (1:500, Jackson Immuno Research, West Grove, PA, USA) for 1 h at room temperature. After washing with PBS, the cells were covered with VECTASHIELD mounting medium (Vector Laboratories, CA, USA) with 40, 6-diamidino-2-phenylindole (DAPI). The sections were observed using a BZ-X 800 (KEYENCE, Osaka, Japan). The numbers of DCX-positive cells, CD31-positive microvessels, and CD31/Ki67 double positive cells in the indicated region were counted and quantified by an investigator who was blinded. Four serial sections spaced 400-μm apart (1.10 to − 0.1 mm from the bregma) were selected from each animal^30^. The numbers of DCX-positive cells, CD31-positive microvessels and CD31/Ki67 double positive cells were counted and averaged from 4 sections of each animal. Sections were incubated with the same primary antibodies and imaged under the same conditions by a blinded investigator. Negative control staining without primary antibodies resulted in no signal. The antibodies used in this study are listed in Table S3.

### Quantitative real-time PCR

To assess the acute effects of the LIPUS therapy in the MCAO model in mice, real-time PCR analysis was performed on day 3 after MCAO. Total RNA was extracted from the ipsilateral striatum and isolated using the RNeasy universal kit (Qiagen, Hilden, Germany) in accordance with the manufacturer’s protocol. After reverse transcription, real-time PCR was performed using SYBR Premix Ex Taq II (Takara-Bio Inc.) and a CFX96TM Real-Time system C1000TM Thermal Cycler (Bio-Rad Laboratories Inc., California, USA). The number of transcripts was normalized to GAPDH as a housekeeping gene. Melting curves were run to ensure amplification of a single product. The primers used in this study are listed in Table S4.

### Western blot analysis

To assess the acute effects of the LIPUS therapy in the MCAO model in mice, Western blot analysis was performed on day 5 after the procedure. The brain was perfused with PBS and cut into 4 sections by 3 blades that are 2-mm apart; the second rostral section, including the ischemic core, was collected. The protein extracted from ipsilateral striatum was used for further Western blot analysis. Western blot analysis was performed on day 5 after MCAO. The tissue was homogenized using Tissue Protein Extraction Reagent (Thermo-Fisher Scientific, Waltham, MA, USA) containing a protease inhibitor cocktail (Sigma-Aldrich, MO, USA). The samples were then centrifuged at 15,000 *g* for 20 min at 4 °C, and the supernatants were collected and normalized for protein concentration, which was measured using the BCA Protein Assay Kit (Biotechnology Inc., Rockford, IL, USA). The samples were then separated by SDS-PAGE and transferred to a polyvinylidene difluoride (PVDF) membrane (GE Healthcare, IL, USA). After blocking with Tris-buffered saline (TBS) containing 5% (w/v) skim milk or BSA and 0.05% Tween 20, the membranes were incubated with primary antibodies overnight at 4 °C. The antibodies used in this study are listed in Table S3. The membrane was then incubated in the appropriate secondary antibody (1:5,000 dilution) for 1 h at room temperature. The proteins were visualized using enhanced chemiluminescence (ECL Western Blotting Detection Kit; GE Healthcare, IL, USA) and quantified using ImageJ Software (NIH).

### Statistical analysis

All summary data are presented as the mean ± s.e.m. Although no statistical methods were used to predetermine sample sizes for the studies, our sample sizes were similar to those generally used in the field. Differences between 2 groups were analyzed using an unpaired two-tailed Student’s *t*-test. Data from multiple groups were analyzed using analysis of variance (ANOVA) followed by Tukey’s multiple comparison (GraphPad Prism Software Inc.). Differences with a P-value < 0.05 were considered to be statistically significant.

## Supplementary Information

Supplementary Video 1.

Supplementary Video 2.

Supplementary Information 1.

## Data Availability

Data are available on request by contacting the corresponding author.
