# Supplementary material for: Low-intensity pulsed ultrasound therapy promotes recovery from stroke by enhancing angio-neurogenesis in mice in vivo
Source: Sci Rep. 2021 Mar 2;11:4958. doi: 10.1038/s41598-021-84473-6 (PMC7925563; doi:10.1038/s41598-021-84473-6)
Supplement: Supplementary file 3 — Supplementary Information 1. [file 41598_2021_84473_MOESM3_ESM.pdf]

# SUPPLEMENTAL MATERIALS

# Low-intensity pulsed ultrasound therapy promotes recovery from stroke by enhancing angio-neurogenesis in mice in vivo

Sadamitsu Ichijo, Tomohiko Shindo, Kumiko Eguchi, Yuto Monma, Takashi Nakata, Yoshihiko Morisue, Hiroshi Kanai, Noriko Osumi, Satoshi Yasuda, Hiroaki Shimokawa.

## Supplemental Files

## Figures S1 to S10

## Tables S1 to S4

## Movies S1 to S2

**Fig.S1**

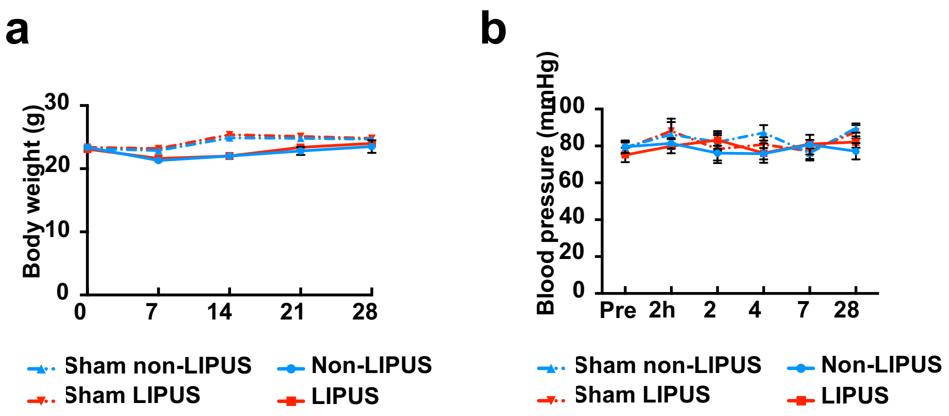


**Figure S1. Body weight and blood pressure**

Graphs showing time-course of body weight **(a)** and mean blood pressure **(b)** measured in the indicated groups (n=10 per group).

**Fig.S2**

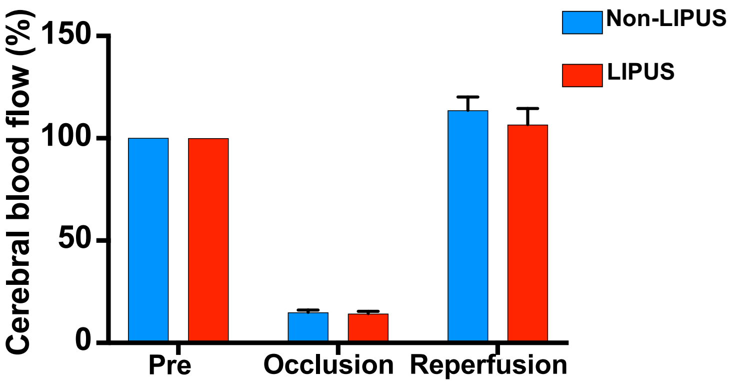


**Figure S2. Cerebral blood flow during MCAO**

Cerebral blood flow was determined by laser Doppler flowmetry (n=10 per group).

**Fig.S3**

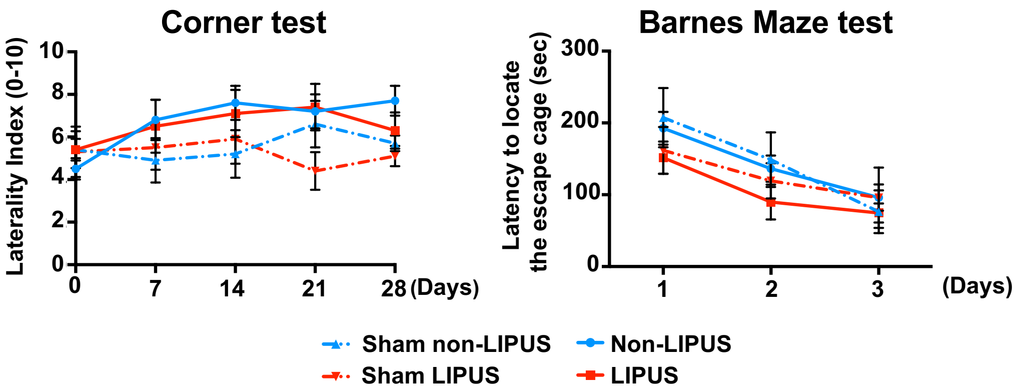


**Figure S3. Results of behavioral tests**

Results of corner test and barnes maze test.

**Fig.S4**

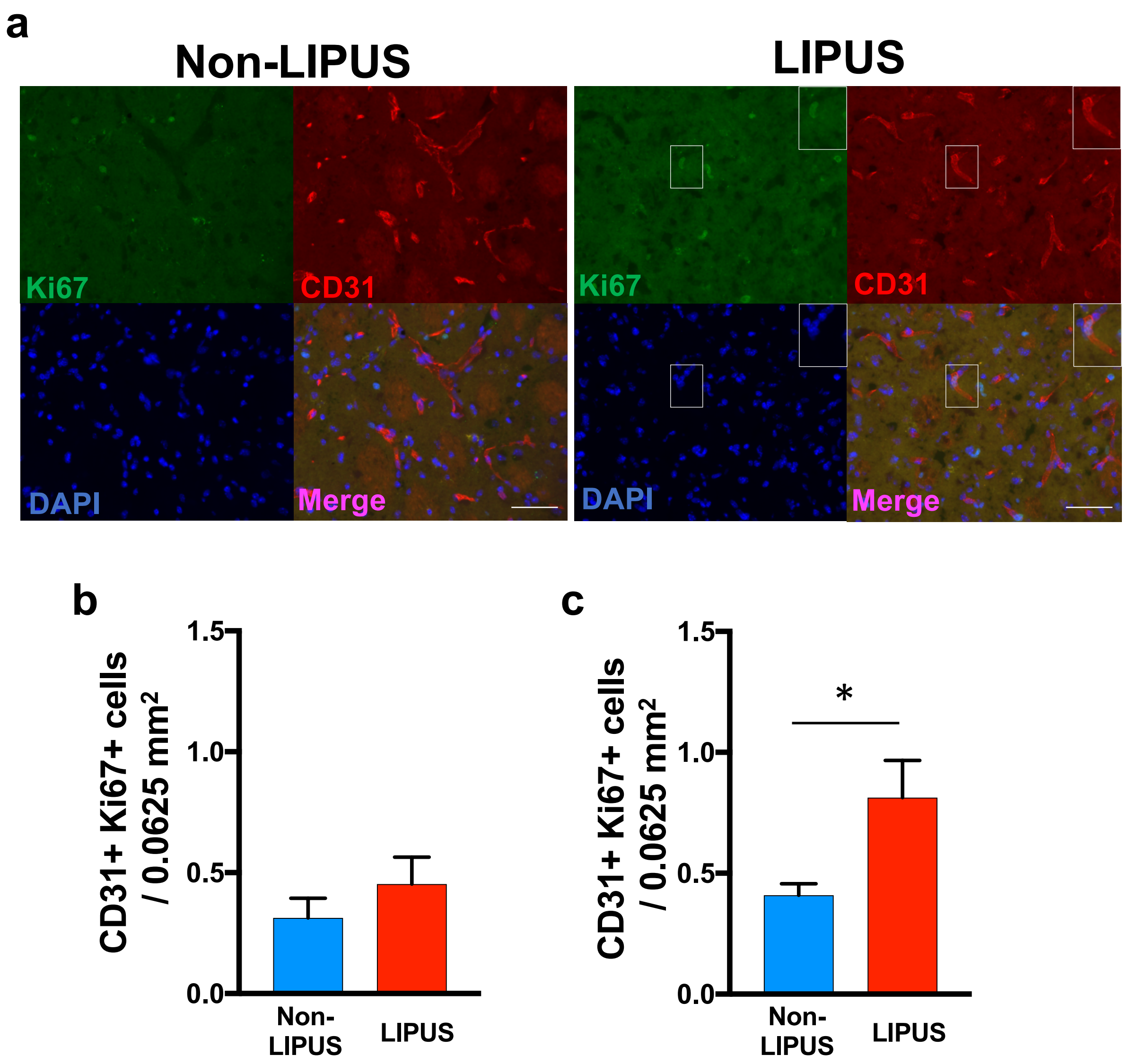


**Figure S4. Effects of the LIPUS therapy on angiogenesis after MCAO**

**a**, Representative images of double immunostaining of Ki67 and CD31 at 500-750 μm from the SVZ on day 7. The scale bar represents 50 μm. Quantification of Ki67+/CD31+ cells at 250-500 μm (**b**) and 500-750 μm (**c**) from the SVZ on day 7 (n=8 per group).

**Fig.S5**

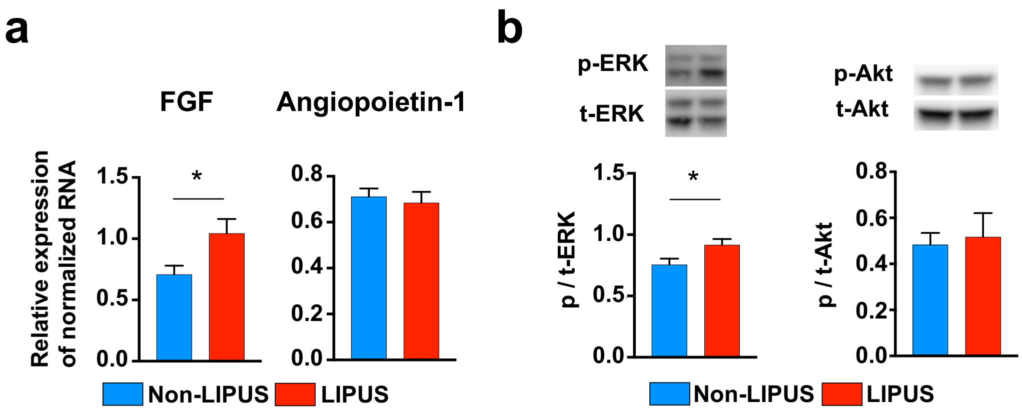


**Figure S5. Effects of the LIPUS therapy on molecular expression after MCAO**

**a**, RT-qPCR analysis of FGF and angiopoietin-1 at day 3 (n=14 per group). **b**, Western blot analysis of ERK1/2 phosphorylation and Akt phosphorylation at day 5 (n=10 per group). **P*<0.05 (Student's *t*-test). Uncropped blots are shown in **Fig. S10**.

**Fig.S6**

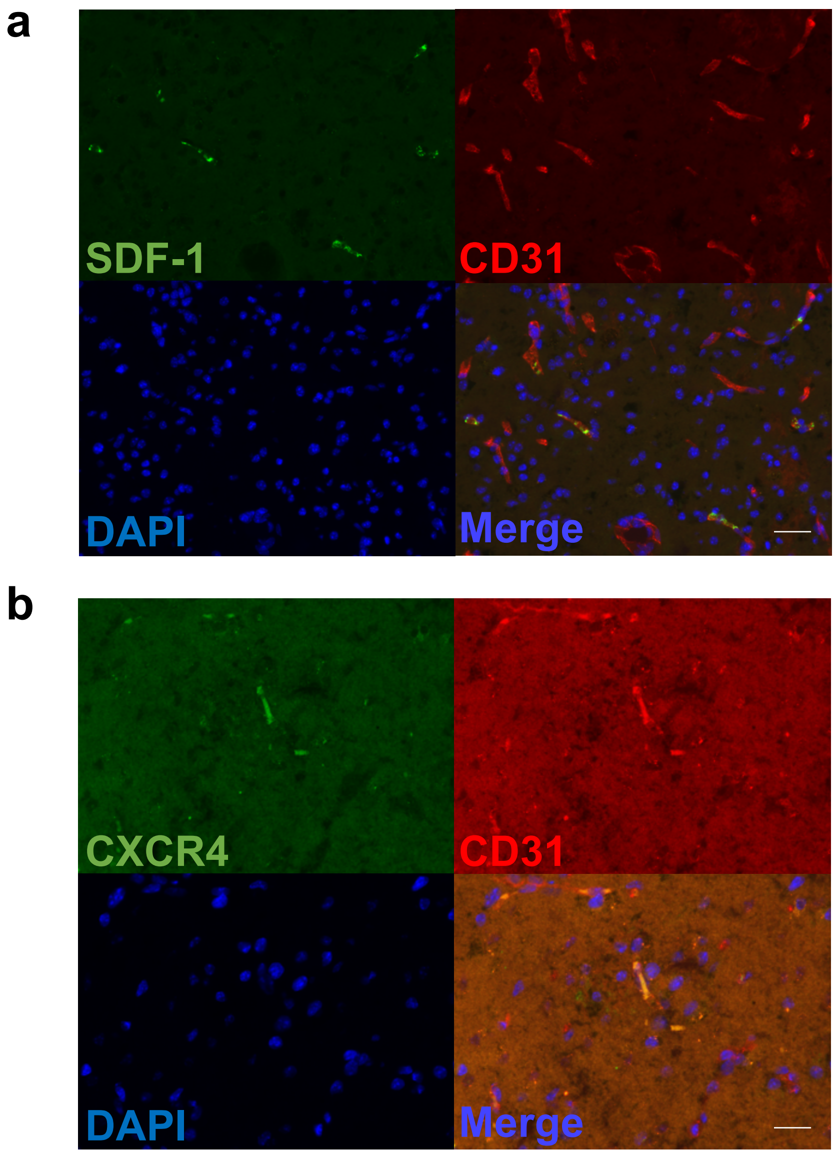


**Figure S6. Expression of SDF-1 and CXCR4 by CD31 positive cells**

**a**, Representative images of double immunostaining for SDF-1 and CD31 in the ipsilateral striatum on day 7. **b**, Representative images of double immunostaining for CXCR4 and CD31 in the ipsilateral striatum on day 7. The scale bar represents 20 µm.

**Fig.S7**

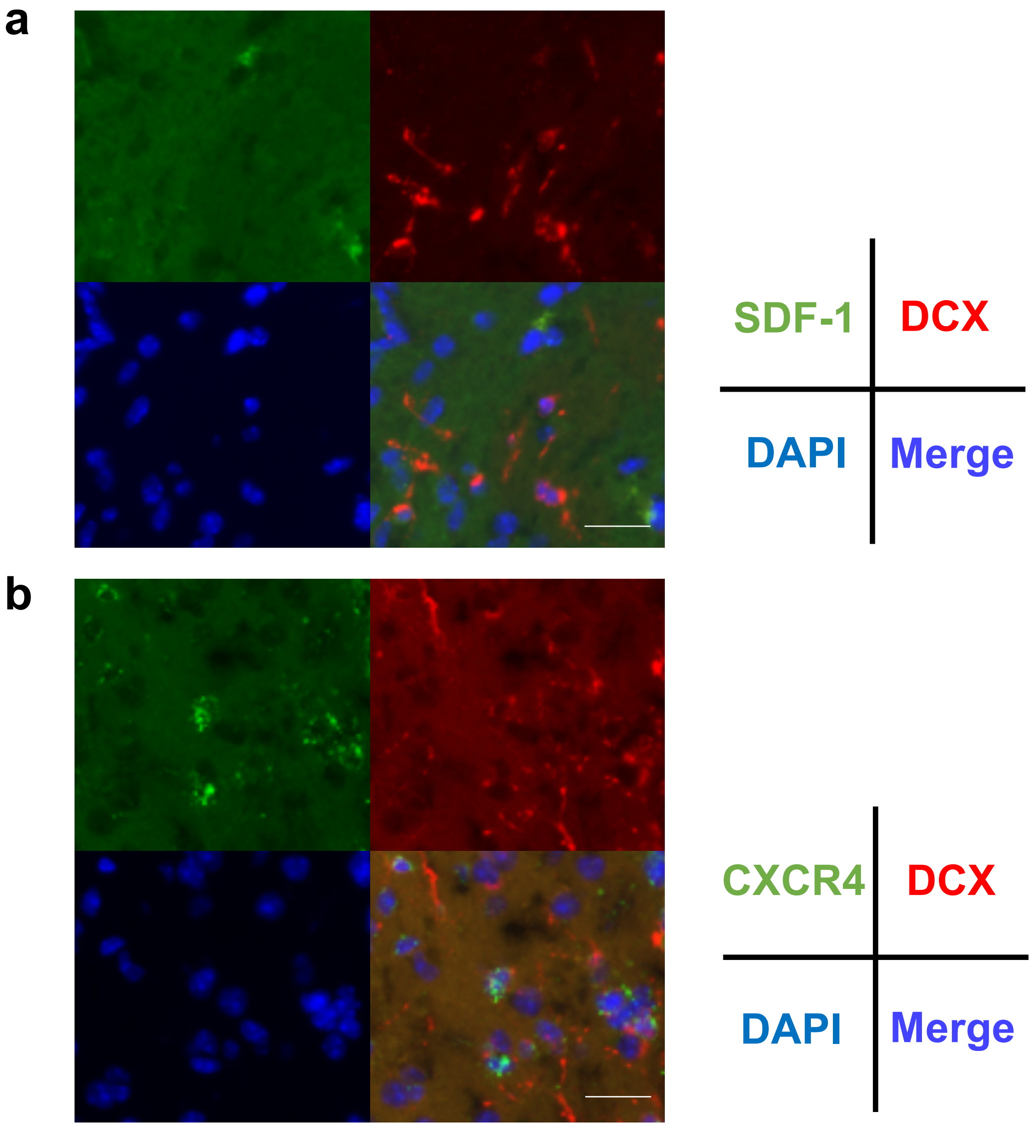


**Figure S7. Expression of SDF-1 and CXCR4 by DCX positive cells**

**a**, Representative images of double immunostaining of SDF-1 and DCX in the ipsilateral striatum on day 7. **b**, Representative images of double immunostaining of CXCR4 and DCX in the ipsilateral striatum on day 7. The scale bar represents 20 µm.

**Fig.S8**

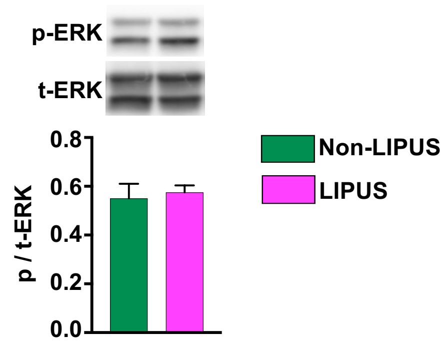


**Figure S8. Effects of the LIPUS therapy after MCAO in eNOS$^{-/-}$ mice**

Western blot analysis of ERK1/2 phosphorylation at day 5 (n=6 per group). Uncropped blots are shown in **Fig. S10**.

**Fig.S9**

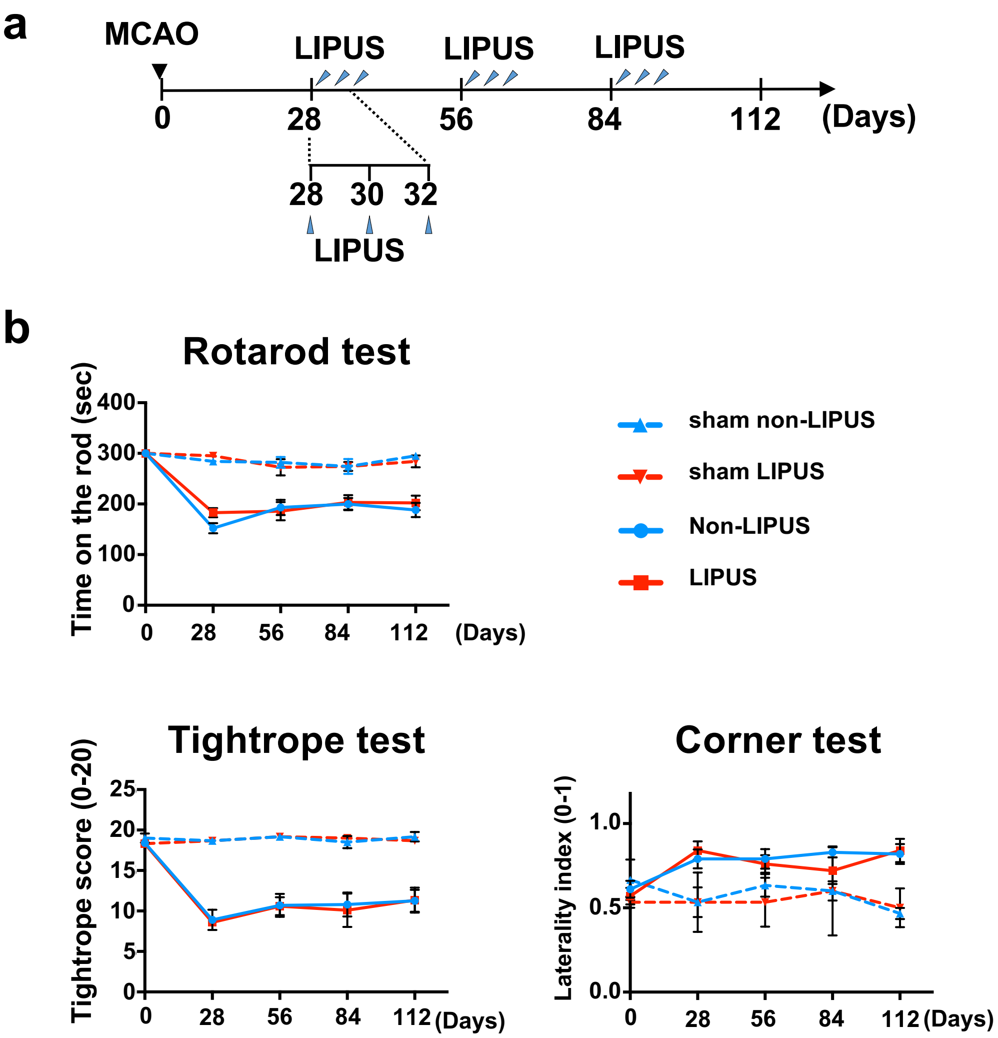


**Figure S9. Effects of the LIPUS therapy on neurological function after MCAO in the chronic phase**

**a**, The timeline of the experiment. **b**, Rotarod, tightrope, and corner tests were performed on days 28, 56, 84, and 112 following MCAO or sham surgery; n=3 (sham), n=10 (MCAO).

**Fig.S10**

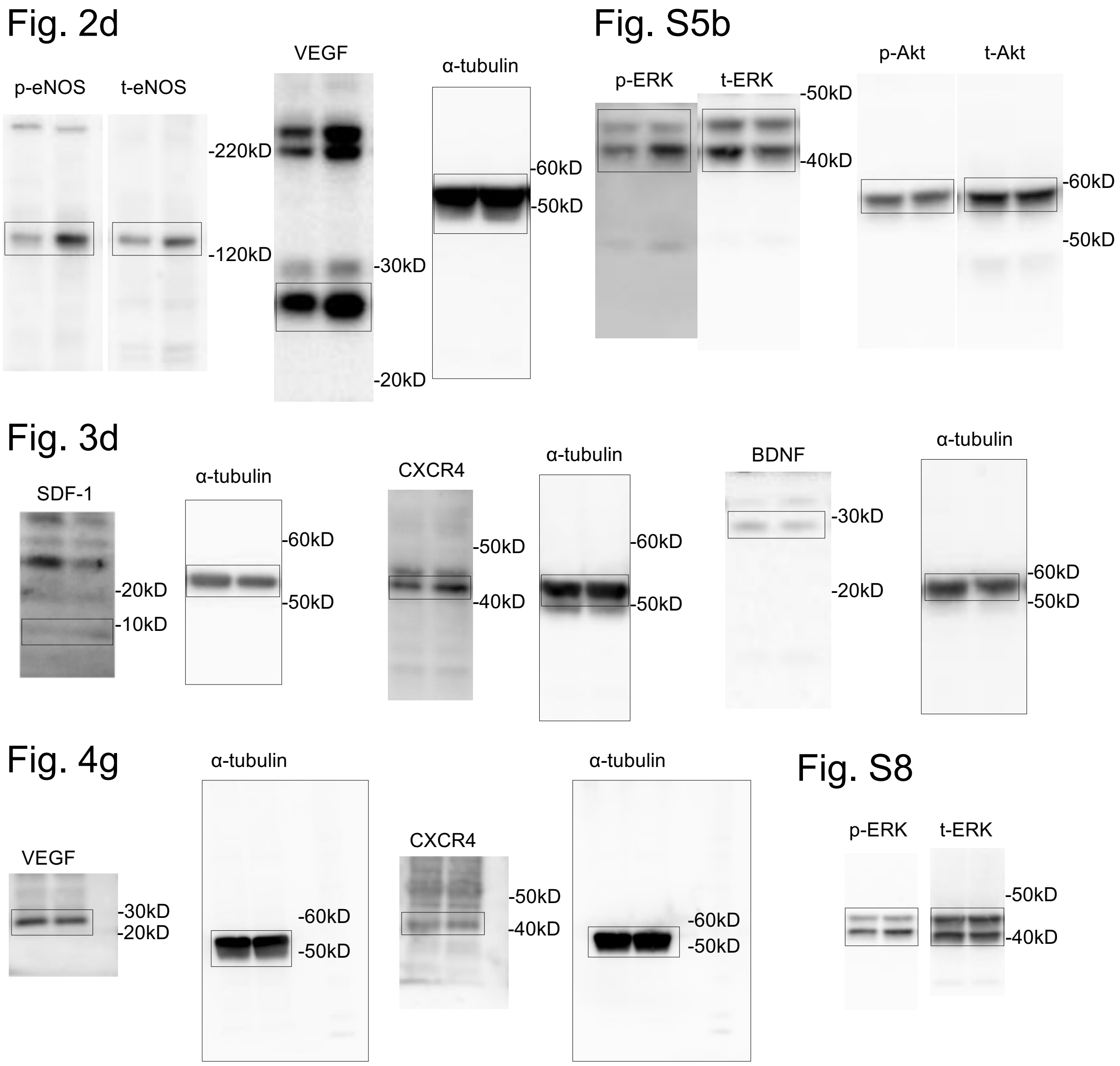


**Figure S10. Uncropped images of Western blots**

The uncropped blots are shown in the main and supplemental figures. Boxed areas indicate the cropped regions shown in the respective figure.

# Tables S1 to S4

**Table 1. Animal number (Survival/total) in each group**

| Group | Behavioral tests (Day 28) | Behavioral tests (Day 112) | IF | CV | RT-PCR | WB |
|---|---|---|---|---|---|---|
| Sham+Non-LIPUS (WT) | 10/10 | 10/10 | | 3/3 | | |
| Sham+LIPUS (WT) | 10/10 | 10/10 | | 3/3 | | |
| MCAO+Non-LIPUS (WT) | 10/14 | 10/14 | 24/31 | 8/12 | 14/19 | 10/15 |
| MCAO+LIPUS (WT) | 10/13 | 10/13 | 24/32 | 8/11 | 14/18 | 10/13 |
| MCAO+Non-LIPUS (eNOS$^{-/-}$) | 6/17 | | 6/16 | 6/15 | | 6/15 |
| MCAO+LIPUS (eNOS$^{-/-}$) | 6/18 | | 6/15 | 6/15 | | 6/13 |
| | 52/82 | 40/47 | 60/94 | 34/59 | 28/37 | 32/56 |
| | | | | | Total: 375 | |

IF: immunofluoro staining; CV: cresyl violet staining; RT-PCR: real time PCR; WB: western blotting; WT: wild type. c

**Table S2. Assessment of tightrope test performance**

| Score | Time (s) | Platform arrival |
|---|---|---|
| 20 | 1-6 | + |
| 19 | 7-12 | + |
| 18 | 13-18 | + |
| 17 | 19-24 | + |
| 16 | 25-30 | + |
| 15 | 31-36 | + |
| 14 | 37-42 | + |
| 13 | 43-48 | + |
| 12 | 49-54 | + |
| 11 | 55-60 | + |
| 10 | 55-60 | - |
| 9 | 49-54 | - |
| 8 | 43-48 | - |
| 7 | 37-42 | - |
| 6 | 31-36 | - |
| 5 | 25-30 | - |
| 4 | 19-24 | - |
| 3 | 13-18 | - |
| 2 | 7-12 | - |
| 1 | 1-6 | - |
| 0 | 0 | - |

Tightrope test results were assessed according to both time on the rope (in sec) and platform arrival ("+" for arrival and "-" for non-arrival). The scores ranged from 0 (minimum) to 20 (maximum).

**Table S3. Antibodies used for Western blot analysis (WB) and immunofluorescence (IF)**

| Antibody | Application | Dilution | Manufacturer | Cat. no |
|---|---|---|---|---|
| Doublecortin (DCX) | IF | 1:500 | Abcam | ab18723 |
| Doublecortin (DCX) | IF | 1:50 | Santa Cruz | Sc-271390 |
| CXCR4 | WB | 1:1000 | Abcam | ab124824 |
| CXCR4 | IF | 1:200 | Abcam | ab124824 |
| BDNF | WB | 1:1000 | Abcam | ab108319 |
| CD31 | IF | 1:500 | MERCK | MAB1398Z |
| CD31 | IF | 1:500 | Dianova | DIA-310 |
| Ki67 | IF | 1:200 | Invitrogen | 14-5698-82 |
| SDF-1 | WB | 1:1000 | Abcam | ab9797 |
| SDF-1 | IF | 1:100 | R&D Systems | MAB350 |
| Phospho-eNOS | WB | 1:1000 | Cell signaling | 9571 |
| eNOS | WB | 1:1000 | BD Biosciences | 610296 |
| VEGF | WB | 1:1000 | Santa Cruz | Sc-7269 |
| α-tibulin | WB | 1:10000 | Sigma | T5168 |
| phospho-ERK1/2 | WB | 1:1000 | Cell Signaling | 9106 |
| total-ERK1/2 | WB | 1:1000 | Cell Signaling | 9102 |
| phospho-Akt | WB | 1:1000 | Cell Signaling | 9271 |
| total-Akt | WB | 1:1000 | Cell Signaling | 9272 |

**Table S4. Primers used for gene expression analysis**

| | Primer sequences for PCR | |
|---|---|---|
| Gene | Forward primer (5’>3’) | Reverse primer (3’>5’) |
| *SDF-1* | 5’CAGAGCCAACGTCAAGCACAC | 5’TTAATTTCGGGTCAATGCACAC |
| *CXCR4* | 5'CCATGGAACCGATCAGTGTG | 5'GCCGACTATGCCAGTCAAGAA |
| *BDNF* | 5’TCAAGTTGGAAGCCTGAATGAAT | 5’CTGATGCTCAGGAACCCAGGA |
| *eNOS* | 5'ATTCTGGCTACACGCAAGACAGA | 5'TCCCGGTAGAGATGGTCCAG |
| *VEGF* | 5’ACATTGGCTCACTTCCAGAAACAC | 5’TGGTTGGAACCGGCATCTTTA |
| *GAPDH* | 5'TGTGTCCGTCGTGGATCTGA | 5'TTGCTGTTGAAGTCGCAGGAG |
| *FGF2* | 5'AAGCGGCTCTACTGCAAGAA | 5'TACCGGTTGGCACACACACTC |
| *Angpt1* | 5’TGATAACCGCCAGCCACAAAG | 5'CCTGTAATGTCGGCACATACCTC |

# Legends to Movies S1 to S2

**Movie S1. Tightrope testing on day 28 (non-LIPUS group).** This movie shows tightrope testing performed by a mouse of the non-LIPUS group on day 28 after MCAO surgery.

**Movie S2. Tightrope testing on day 28 (LIPUS group).** This movie shows tightrope testing performed by a mouse of the LIPUS treated group on day 28 after MCAO surgery.
